# Demographic forecasting of population aging in Greece and Cyprus: one big challenge for the Mediterranean health and social system long-term sustainability

**DOI:** 10.1186/s12961-020-00666-x

**Published:** 2021-02-15

**Authors:** Demetris Lamnisos, Konstantinos Giannakou, Mihajlo (Michael) Jakovljevic

**Affiliations:** 1 Department of Health Sciences, School of Health Sciences, European University Cyprus, Nicosia, Cyprus; 2 Institute of Comparative Economic Studies, Hosei University, Tokyo, Japan; 3 Department of Public Health and Healthcare named after N.A. Semashko, I.M. Sechenov First Moscow State Medical University (Sechenov University), Moscow, Russia; 4 Department of Global Health Economics and Policy, Faculty of Medical Sciences, University of Kragujevac, Kragujevac, Serbia

**Keywords:** Demographic transition, Demographic forecasting, Population aging, Bayesian hierarchical models, Greece, Cyprus

## Abstract

**Background:**

With an increasing aging population and a lower ratio between the active and the dependent population, population aging is considered a global social and health challenge, associated with increased demand in health care needs and social pension. This study projects the Greek and Cypriot population to guide future planning of social and health policies and services.

**Methods:**

The total population by sex and age groups, Total Fertility Rate (TFR), life-expectancies at birth and Potential Support Ratio PSR (persons aged 20–64 years per person 65+ years) are projected probabilistically by the year 2100 using Bayesian hierarchical models and United Nations’ population data for Greece and Cyprus from the period of 1950 to 2015.

**Results:**

The TFR is projected to be around 1.5 children per woman in 2050 and around 1.75 in 2100 for both countries, with all values of prediction intervals being around or below the Replacement level fertility. PSR is expected to decrease remarkably and be 2.5 in 2050 and 1.6 in 2100 for Cyprus while for Greece it will be around 1.5 for both years 2050 and 2100. Life-expectancy is expected to increase to 84 years for men and 87 years for women in 2050 and 90 years for men and 94 years for women in 2100 for both countries. The share of the population aged 65 years and over is projected to increase in both countries and be the one third of the population by 2100.

**Conclusions:**

Greece and Cyprus will acquire the characteristics of an aging population, putting a significance pressure on the social and health systems of both countries. Both countries should reform their social and health policy agenda to confront population aging and its consequence. They should adopt fertility incentives and family policies to increase fertility and migrants’ inclusiveness policies to improve the demographic structure and the economic activity. The national health systems should promote prevention strategies at the primary health sector and promote healthy aging while health research policy should aim to promote research in innovative technologies and digital health to create assistive technology for self-care and greater independence of older people.

## Introduction

Population aging is a universal phenomenon which is considered one of the most important issues throughout most of the world. Advances in nutrition, medicine, childhood survival, socioeconomic development and public health have substantially increased longevity as mortality and morbidity rates from the most virulent infectious diseases and, to some extent, from noncommunicable diseases are reduced [1]. Worldwide, life-expectancy at birth has increased from 52.9 years in 1950 to 75.6 years in 2017 for women and from 48.1 years to 70.5 years for men, respectively [2]*.* At the same time, estimations in 2017 show that almost half of the countries or areas with at least 90,000 inhabitants have fertility levels below that required for the long-term replacement of the population [3]. Hence, the continuous decline in fertility rates as a consequence of the evolvement of interpersonal relationships and the absorption of women into the labour markets in combination with the increase in life-expectancy are causing a significant shift of global population age structure to a higher proportion of older people [4–6]. Population aging and life-expectancy continues to increase worldwide as both mortality rates at older ages and fertility rates decrease. In fact, according to The United Nations Population Division report, the population aged older than 60 years nearly tripled between 1950 and 2000 and will increase to more than two billion in 2050, representing 22% of the world population [7].

Undoubtedly, this transition comes with an unprecedented set of challenges, considering that this growing age group has greater needs in terms of health care, economic, social, cultural and other aged-care services [8, 9]. The lower ratio between the active and the dependent population, and the higher demands of this age group for health care, social care, and social pensions pose a significant global challenge for the long-term financial sustainability of national health systems and public social and health insurance funds [6, 8, 10–12]. Population aging is associated with numerous policy challenges, and the fact that we are not able to consider previous historical episodes for guidance on how this demographic upheaval will unfold creates even more difficulties in public health systems. Projections of countries’ future populations is a vital tool that can be used by governments, international organizations and the private sector to understand future population dynamics of the society, plan for the future, and inform decision-making [13, 14]. Despite its uncertainties, population projection is essential in pension planning, preparing health provision, providing future retail services as well as enhancing the education of the population [14].

Europe’s population today is the most aged compared with other regions, with 25% of the population aged 60 years or over in 2017 [3]. Like other rapidly aging nations, the impact of population aging in Greece and Cyprus will become progressively clear in the next few decades. The demographic aging problem in Greece and Cyprus is also amplifying by the recent economic crisis that occurred in 2008 for Greece and in 2012 for Cyprus and caused a significant reduction in birth rates among young women [15–17]. Thus, it is critical to plan ahead for the future, to ensure adequate health care, financial, and other services suitable for an aging population. The aim of this study was to project the Greek and Cypriot populations using Bayesian hierarchical models. We choose to study simultaneously both countries as they are both members of the European Union and form part of a Greek-speaking community with shared family, social and cultural aspects. To our knowledge, this is the first nationwide population projection on such a detailed level for both Cyprus and Greece to guide future planning of public health and social policies and systems.

## Material and methods

### Data sources and data

The United Nation’s data for all countries from the period of 1950 to 2015 were used in this study [3]. These data includes the total population and distribution by gender and age-groups (with 5-year age bands) of each country for the time period between 1950 to 2015, the Total Fertility Rate from 1950 to 2015, the life-expectancy at birth and mortality for each age-group from the period of 1950 to 2015 as well as international migration.

### Statistical methods

Most methods for predicting population *P* in country c at time period *t* are based on the demographic balancing equation, namely:

$$Pc,t = Pc,t - {1} + Bc,t - Dc,t + Mc,t,$$

where *B* denotes the number of births, *D* denotes the number of deaths and *M* denotes net international migration. In most applications this equation is solved deterministically using the cohort component method [18], which decomposes it in to age-specific and sex-specific components. The United Nations Population Division has used the cohort component model to project deterministically a country’s population by age and sex in future time periods *t* > 0, and requires the following inputs: sex-specific and age-specific population estimates at the initial time *t* = 0, projections of future Total Fertility Rates (TFR), projections of sex ratio at birth, projections of female and male life-expectancies, historical data on sex-specific and age-specific death rates (for *t* ≤ 0), historical data on fertility distribution by age (for *t* ≤ 0), and projections of future sex-specific and age-specific net international migration. In the above inputs, the TFR is defined as the average number of live births that a hypothetical cohort of women would have at the end of their reproductive period if they were subject during their whole lives to the fertility rates of a given period and if they were not subject to mortality [19]. The TFR is expressed as live births per woman. The life-expectancy at birth is defined as the average number of years of life expected by a hypothetical cohort of individuals who would be subject during all their lives to the mortality rates of a given period and it is expressed as years [19].

In each time period *t*, a projection of the fertility distribution by age is obtained using historical data [20]. Then, this distribution is used to convert the TFR to age-specific fertility rates in time period *t*. Using the historical data on death rates, life-expectancy is converted to age-specific mortality rates using a variant of the Lee-Carter method [21]. Finally, the cohort component method is applied. Methods for probabilistic projection of the TFR and life-expectancy have been recently developed [22, 23]. Raftery et al. (2012) [13] introduces a way to combine TFR and life-expectancy in to overall probabilistic population projections. Their method is based on simulating a large number of trajectories of future values of TFR [19], then to simulate an equal number of trajectories of life-expectancy [24], and finally to convert each of the trajectories in to a future trajectory of all sex-specific and age-specific populations, using the current United Nations’ methodology as described above.

First, the package bayes TFR [19] of the R software (R Core Team, 2016) was used to probabilistically project the TFR and life-expectancy for all countries using Bayesian hierarchical models [22, 23]. This package implements Markov chain Monte Carlo methods to sample many trajectories from the joint predictive distribution of TFR and female and male life-expectancies for all countries and all future time periods up to 2100. Next, the statistical package bayesLife [25] was applied, which computes the trajectories of age-specific and sex-specific mortality rates using the trajectories of life-expectancies already produced in the previous step, and the age-specific mortality rates using the trajectories of Total Fertility Rates already produced in the previous step. Finally, package bayesPop was used, which utilizes the cohort component model and all sample trajectories to compute the future trajectories of population by gender and age for all countries and future time periods up to 2100 [26].

The population trajectories are a sample from the joint population predictive distribution and these sample trajectories are used to derive the predictive median of any future population quantity of interest as well as its 80% and 95% Prediction Interval (PI). The population quantities of interest in this study were the total population over 65 years old in Greece and Cyprus for the time period 2020–2100, the life-expectancy at birth for women and men in Greece and Cyprus for the time period 2020–2100, the TFR in Greece and Cyprus for the time period 2020–2100 and the Potential Support Ratio (PSR) (defined as the number of persons aged 20–64 years per person 65 + years) in Greece and Cyprus for the time period 2020–2100.

## Results

**Fig. 1:**
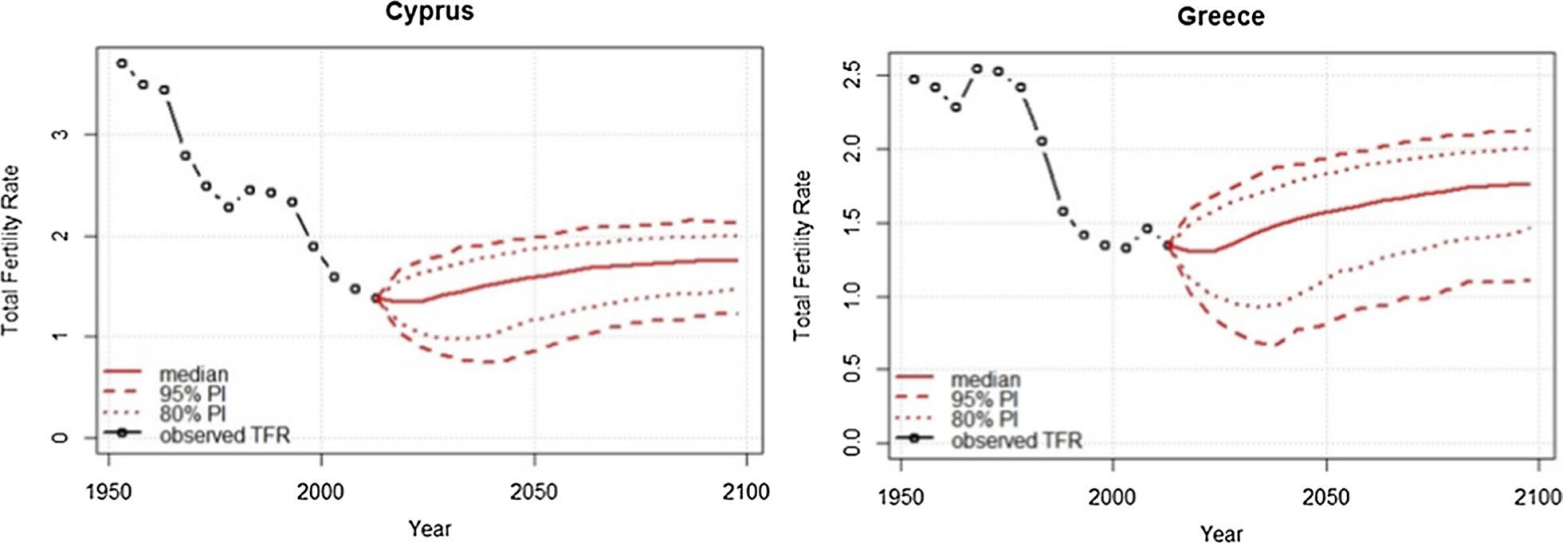
Total fertility rate projected for the time period 2020–2100 for Cyprus and Greece. *PI* prediction interval

Figure 1 shows the future projection of TFR for the time period 2020–2100 for Cyprus and Greece. The solid red line shows the median trajectory of TFR. The TFR in Cyprus decreased from around four children per woman in the 1950s to 1.4 in the period 2005–2010. The TFR is projected to have a slight increase to 1.53 children per woman (95% PI 0.84, 1.97) by 2050 and 1.75 (95% PI 1.23, 2.12) by 2100, with all values of prediction intervals being around or below the replacement-level of fertility which is the TFR of 2.1. As regards to the TFR for Greece, this has followed a strong downward trend from 2.5 children in 1950 to 1.4 in 2005–2010 and it is projected to have a slight increase to 1.50 children per woman (95% PI 0.79, 1.93) by 2050 and 1.74 (95% PI 1.12, 2.13) by 2100, with all values of prediction intervals being around or below the replacement-level of fertility.

**Fig. 2:**
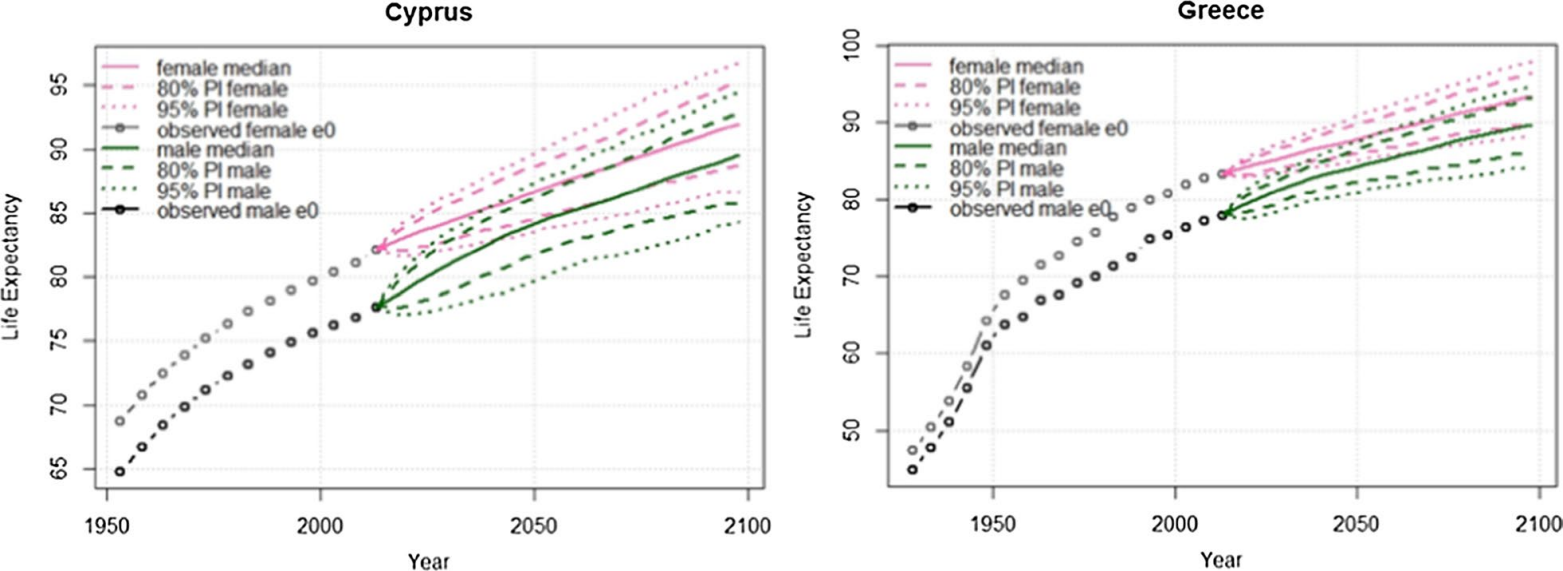
Life expectancy projected for the time period 2020–2100 for Cyprus and Greece. *PI* prediction interval

Figure 2 displays the projections of female and male life-expectancies for the time period 2020–2100 for Cyprus and Greece. female and male life-expectancies in Cyprus were 68.5 and 65 in 1950 and increased to 82.2 and 77.7 in 2010. The female and male life-expectancies is predicted to steadily increase and reach the value of 86.4 (95% PI 83.4, 89.5) and 83.9 (95% PI 79.4, 87.0) in 2050, and 92.0 (95% PI 86.6, 96.8) and 89.4 (95% PI 84.3, 94.6) in 2100. A similar trend in life-expectancy is also observed for Greece. female and male life-expectancies in Greece were 65 and 61 in 1950 and increase to 83.3 and 78 in 2010. The life-expectancy is predicted to increase and reach the age of 87.6 years for women (95% PI 84.8, 90.6) and 84.0 for men (95% PI 80.7, 87.1) in 2050, and 93.3 for women (95% PI 88.3, 98.0) and 89.7 for men (95% PI 84.0, 94.7) in 2100.

**Fig. 3:**
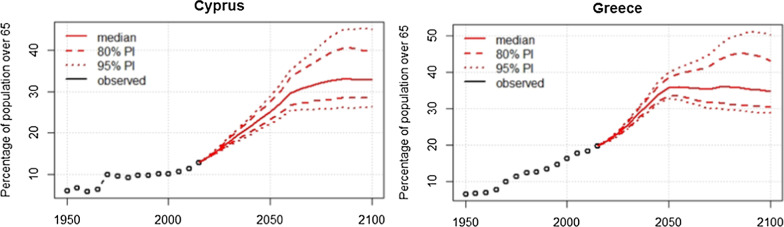
Percentage of population aged over 65 years projected for the time period 2020–2100 for Cyprus and Greece. *PI* prediction interval

The future projection of the percentage of the population over 65 for the time period 2020–2100 for Cyprus and Greece is presented in Fig. 3. The percentage of the population aged over 65 years in Cyprus is projected to increase from 12% in 2010 to 25% (95% PI 21.0, 29.2) in 2050 and 33% (95% PI 27.0, 44.0) in 2100. Likewise, in Greece, the percentage of the population aged over 65 years is increasing from 20% in 2010 to 36% (95% PI 33, 40) in 2050 and 35% (95% PI 29, 50) in 2100.

**Fig. 4:**
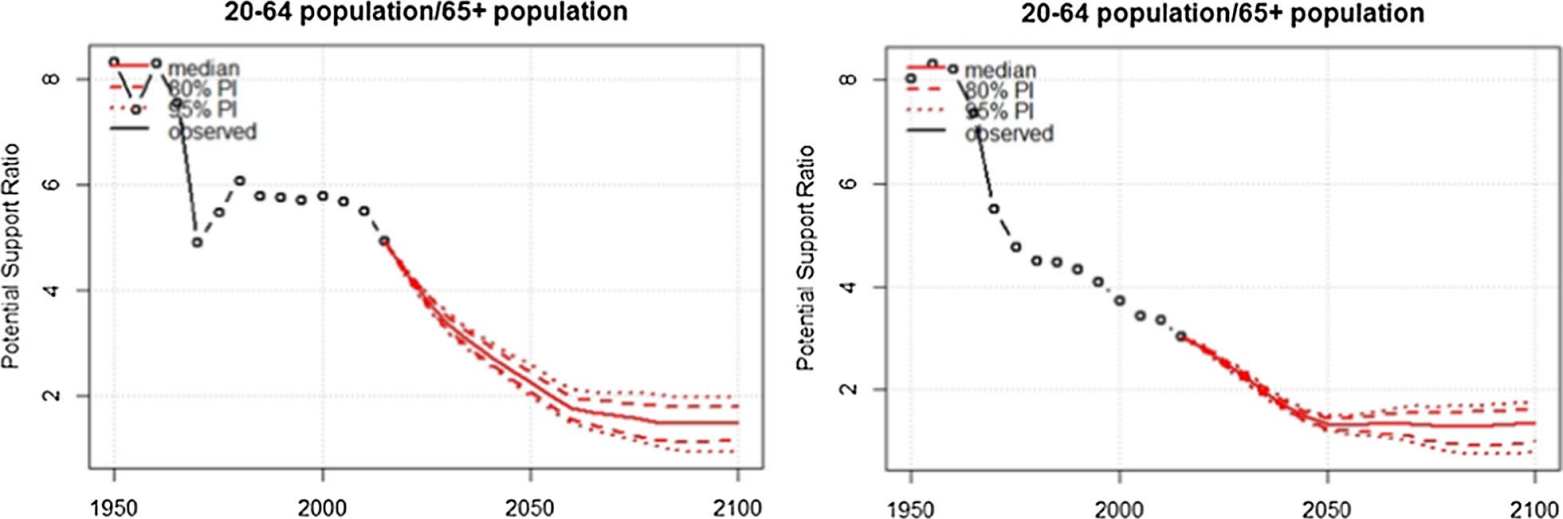
Potential Support Ratio (population aged 20–64 years∕population aged over 65 years) for the time period 2020–2100 for Cyprus and Greece. *PI* prediction interval

Figure 4 presents the future projection of PSR for the time period from 2020 to 2100 for Cyprus and Greece. The PSR in Cyprus decreased from 8.5 people age 20–65 per one older person aged 65 or older to 5 in 2010. The PSR is projected to decrease to 2.5 people (95% PI 2.0, 2.8) by 2050 and 1.6 people (95% PI 1.4, 2.0) by 2100. As regards to the PSR for Greece, this has followed a strong downward trend from eight people age 20–65 years per one older person aged 65 years or older in 1950 to three people in 2010 and is projected to decrease further to 1.5 people (95% PI 1.4, 1.6) by 2050 and 1.5 (95% PI 0.8, 1.8) by 2100, with all values of the prediction interval being below two people aged 20–65 years per one older person.

## Discussion

In this study we use a Bayesian method for probabilistic population projection to provide a predictive distribution of the future population for Cyprus and Greece. The probabilistic projection methods applied in this study are those used by the Population Division of the Department of Economic and Social Affairs of the United Nations Secretariat to produce the official United Nations population estimates and projections [27]. The results of this study suggest an aging society in Cyprus and Greece with an increasing aging population, a strong downward trend of fertility and a concerning Potential Support Ratio below 2.

The results of this study showed that both countries share quite similar fertility rates, with fertility rates declining from about three children per woman in 1950 to 1.5 children in 2050 and 1.75 in 2100. All values of fertility rates are below the replacement level. On the other hand, female and male life-expectancies will increase from about 65 years in 1950 to 87 and 84 years for women and men in 2050 and to 93 and 90 years for women and men in 2100. The low fertility and the increase in longevity and life-expectancy has as a consequence the demographic transition to an aging population and a shrinkage of working, young-age people [4, 6]. Indeed, the share of the population aged 65 years and over is projected to increase in both countries during 2020–2100 while the ratio of capable labour force to older individuals is predicted to decrease dramatically in the same period. Our projections indicated that one third of the population will be over 65 years old in 2100 for both countries while the Potential Support Ratio will be close to 1.5 persons meaning that 1.5 working-age people (20–65 years) will correspond to one older citizen.

This observed pattern of population aging in Greece and Cyprus is consistent with that of the whole of Europe which was the first region globally that entered the demographic transition, having begun the shift to lower fertility and increasing life-expectancy by the late 19th and early twentieth centuries in almost all of its countries [28]. Europe is projected to be the most aged region in the coming decades with one third (34%) of the population projected to be over 60 years old in 2050, following by North America (28%), Latin America and the Caribbean (25%), Asia (24%) and Oceania (23%) [6]. According to the projections of the United Nations World Population Prospects [29], one third (30%) of European population will be aged over 65 years old in 2100. Although this forecast is a bit lower than those of Greece (35%) and Cyprus (33%), it is also indicating the concerning pattern of population aging in Europe. The Total Fertility Rate in Europe is projected to be 1.75 by 2100 which is similar to the values for Greece and Cyprus and below the replacement level. Therefore, the observed pattern of population aging in Greece and Cyprus is aligned to that of the whole of Europe.

Greece and Cyprus should reform their social and health policy agenda to confront the demographic transition to population aging. Specific policies to increase fertility are the adoption of fertility incentives, family policies and fertility care policies. These policies are general ones and adopted from all countries aiming to increase fertility. Specific measures of fertility incentives and family policies include baby bonuses, family allowances, maternal, paternal, and parental leave, tax incentives, and flexible work schedules. France is one successful example of a European country that manages to increase fertility [30, 31] and could be a good example for all other European countries. This country’s success is partly attributed to the benefits and facilities provided as well as direct payments to families for the care of children. Moreover, France has adopted early childhood strategies which can be categorized in to three groups: (a) increased child-care facilities (nurseries) and better financial support for them, (b) increased benefits to provide partial cover of the cost of child care by registered childminders, and (c) paid parental leave for a parent who withdraws, either partly or entirely, from the labour market [30, 31]. Fertility care policies encompasses the prevention, diagnosis and treatment of infertility and it is indirectly contributing towards improving fertility. Therefore, fertility care should be prioritized in the national public health and social policy agenda of Greece and Cyprus.

Migrants and refugees’ policies that aim to achieve migrants’ integration and inclusiveness are critical to confront the demographic transition to population aging in Greece and Cyprus. These policies are country-specific because Greece and Cyprus are experiencing a large inflow of migrants and refugees, including economic migrants, the last 5 years as a consequence of wars and conflicts. Migrants and refugees, including economic migrants, can bring significant benefits to the countries that host them, including improved demographics and heightened economic activity and productivity. Both countries should aim to familiarize migrants and refugees with the culture of the country and assist them to settle into the community [32]. Greece and Cyprus should aim to engage multiple stakeholders, such as city governments, public health authorities, local businesses, community and civil sector organizations, and schools for joint initiatives on immigrant integration that include migrants, both financially and socially [32]. Furthermore, the adaptation of health (and other) services to make them inclusive and migrant-friendly not only ensures that migrants’ health problems are adequately treated but also has positive impacts on the quality, efficiency, and effectiveness of the services for all in the society [33].

The ever-growing older age cohorts which are expected to account for a substantial proportion of total population in the future and the ever-narrowing of labour-force participants raise serious concerns about the long-term financial sustainability of the welfare and retirement system. The funds for the social support and welfare system of older people will inevitably decrease as the tax base of work-capable young employees will decrease as well. An aging population inevitably creates difficult challenges to the fiscal integrity of public and private pension systems due to a lower percentage of working individuals contributing to the system and higher percentages of benefits recipients associated with increased longevity [34]. At the same time, as the number of older populations increase, they will become larger and politically stronger, generating difficulties in the implementation of certain policies such as the reduction of health or pension benefits. A common policy response to address the financial unsustainability of the pension systems and the diminished economic growth due to the increasing life-expectancy is the extension of retirement age [8]. Another option is the development of plans for a gradual retirement. Those plans have the retired population care for the older population and allow older individuals to reduce progressively their working hours yet remain in the workforce and pay taxes until a later age [5]. Health aging policies could also contribute towards maintaining the active involvement of older citizens to society [35–38]. These policies are about creating an enabling environment to permit the maintenance and preservation of mental and physical capacity and functional ability that enable wellbeing in older age [35].

The growing cohort of older citizens increases the demand of health care and challenges the long-term financial sustainability of national health systems. In particular, the growing cohort of older persons poses a great challenge on the sustainability of the newly established 2018 universal health care system in Cyprus. Inevitably the older population is much more likely to experience multiple, coexistent, and interrelated health problems, and this multimorbidity corresponds to substantial health care needs and loss to national productivity [34, 39]. The national health systems of Greece and Cyprus should promote prevention strategies, including the education and awareness of certain diseases and their corresponding risk factors as well as early screening for several chronic diseases. The health systems in both countries should also promote and facilitate family caregiving in cases where this is feasible and provide support to family caregivers. Family caregiving plays a crucial role in delaying and possibly preventing the hospitalization of chronically ill older patients.

Besides the policy aspects of population aging, the role of behaviour change, especially changes that promote healthy aging, were identified as key responses to many of the challenges associated with population aging. Much of the disease burden in the older population could be prevented or delayed if emphasis is given on disease prevention and health promotion by encouraging behavioural-change strategies such as adherence to a healthier diet, reduction of excessive alcohol consumption, smoking cessation, as well as a reduction of sedentary behaviour [8, 34, 40].

The demographic transition towards an aging population demands from the countries that they develop new innovative technologies to create enabling environments for the older population for their well-being and maintenance of quality of life. To this end, Greece and Cyprus health research policy should aim to promote research and innovation in information and communication technologies (ICT) and digital health to create these enabling environments for the older population to allow for self-care and greater independence [6].

### Strengths and limitations

This study provides population projections and future demographic metrics for Greece and Cyprus and highlights the population aging issue which will be faced by both countries in the next few decades. These metrics will be available to policymakers in both countries and give them the opportunity to plan ahead and reform the social and health policies to make long-term, financial sustainable national health, social and pension systems. The population projections and demographic metrics will be the basis for investigating with econometric models the long-term sustainability of health care and retirement systems in both countries.

However, this study has some limitations which are based on the assumptions made by the projections. These projections do not consider the uncertainty about international migration which is expected to be experienced in a considerable effect by Greece, the main point of migrants’ entry to the European Union. This uncertainty about international migration is an important driver of forecast errors for short time horizons (10 to 20 years) and we are expecting not to have a considerable effect on the long-term population estimates. On the other hand, these projections take in to consideration the uncertainty about future overall levels of fertility and mortality which are the main drivers of forecast error for larger horizons (more than 20 years) and the most important determinants of future population trends. Therefore, the population projection data displayed in this paper should be interpreted with some caution.

As soon as the latest revision of the United Nations World Population Prospects becomes available, the empirical data that inform its latest set of estimates will be released online. The applied projection methods in this study could easily incorporate those empirical data into the statistical models to update the population projections. The United Nations World Population Prospects are updated every 2 years (the most recent was in 2019) and includes, among other data, vital registration of births and deaths, international migration statistics and international estimates from the refugee statistics [41]. Therefore, data about the consequence of the recent economic crisis in both countries on fertility and the massive experience of immigrants and refugee’s inflow in Greece will be included in the latest revision of the United Nations World Population Prospects. Those data will be used by the population projection methods implemented in this study and, in this way, the future projections will take in to consideration the effect of economic and social crises in both countries as well as the massive waves of immigration that occurred in Greece in the last decade.

## Conclusions

The population projections in Greece and Cyprus show that there will be a substantial increase in life-expectancy and in the share of the population aged 65 years and over in the next few decades while in the same time there will be a decline in fertility and the ratio of the active to the dependent population. This demographic transition puts significant pressure on the social and health systems of both countries. There is an urgent need for a comprehensive social and health approach to population aging with social and health policies and strategic responses to these imminent challenges to ensure that there is adequate infrastructure across settings to confront the demographic issues and strengthen the promotion of healthy aging.

## Data Availability

The datasets used and/or analysed during the current study are available from the corresponding author on reasonable request.
